# Postoperative complications after Minimally Invasive transCervical oEsophagectomy (MICE) versus Transthoracic Minimally Invasive oEsophagectomy (TMIE): an inverse probability of treatment weighted analysis

**DOI:** 10.1093/dote/doag106

**Published:** 2026-09-22

**Authors:** Justin G A Grootenhuis, Sam Wijers, Gerjon Hannink, Irma Adbegovic, Jasmijn Herruer, Irene F Kramer, Merlijn Hutteman, Sander Ubels, Bastiaan R Klarenbeek

**Affiliations:** Department of Surgery, Radboud University Medical Center, Nijmegen, The Netherlands; Department of Surgery, Radboud University Medical Center, Nijmegen, The Netherlands; Department of Medical Imaging, Radboud University Medical Center, Nijmegen, The Netherlands; Department of Rehabilitation/ speech therapy, Donders Institute for Brain, Cognition and Behaviour, Radboud University Medical Center, Nijmegen, The Netherlands; Department of Otorhinolaryngology (ENT), Radboud University Medical Center, Nijmegen, The Netherlands; Department of Surgery, Radboud University Medical Center, Nijmegen, The Netherlands; Department of Surgery, Radboud University Medical Center, Nijmegen, The Netherlands; Department of Surgery, Radboud University Medical Center, Nijmegen, The Netherlands; Department of Surgery, Canisius Wilhelmina Hospital, Nijmegen, The Netherlands; Department of Surgery, Radboud University Medical Center, Nijmegen, The Netherlands

**Keywords:** anastomotic leakage, esophagectomy, Minimally Invasive transCervical oEsophagectomy, pneumonia, postoperative pulmonary complications

## Abstract

Minimally Invasive transCervical oEsophagectomy (MICE) is a novel technique with radical lymphadenectomy that avoids a transthoracic access, potentially preserving pleural integrity and obviating one lung ventilation, thereby reducing postoperative pulmonary complications. However, its advantages over conventional Transthoracic Minimally Invasive oEsophagectomy (TMIE) remain unclear. The objective of this study was to assess postoperative outcomes after TMIE and MICE. A retrospective cohort study was conducted including patients with resectable esophageal cancer (cT1–4aN0–3M0) who underwent esophagectomy at the Radboudumc, the Netherlands between 2020 and 2024. The primary outcome was the incidence of postoperative complications within 30 days. Inverse probability of treatment weighting (IPTW) was used to account for confounding between the groups. A total of 115 MICE and 172 TMIE patients were included. After IPTW, overall postoperative complications were more frequent after MICE (60.3% vs. 43.9%, RD: 16.4%, 95% CI: 3.3–29.4). This difference was predominantly driven by a higher incidence of recurrent laryngeal nerve palsy (RLNP) (MICE: 30.4% vs. TMIE: 0.0%). No statistical significant difference was observed in pulmonary complications (18.9% vs. 21.8%, RD: −2.9, 95% CI: −17.1 to 12.5) or anastomotic leakage (5.6% vs. 8.1%, RD: −2.4%, 95% CI: −11.5 to 7.3). Although overall postoperative complication rates were higher after MICE, this difference was mainly attributable to an increased incidence of mostly transient recurrent laryngeal nerve palsy, which is inevitably associated with the transcervical approach. Rates of pneumonia and anastomotic leakage were similar between MICE and TMIE. As these data include cases performed during the early experience with MICE, complication rates may improve as experience accumulates across surgical teams. These findings highlight the need for further evaluation and optimization of the MICE procedure prior to broader implementation.

## INTRODUCTION

Esophageal cancer is a highly aggressive malignancy with a poor prognosis, with a five-year overall survival rate of approximately 50% following Transthoracic Minimally Invasive oEsophagectomy (TMIE).^1^^,^^2^ Surgical resection with radical two- or three field lymphadenectomy, typically following neoadjuvant chemoradiotherapy or perioperative chemotherapy, is considered the cornerstone of curative treatment for esophageal cancer in Europe.^3^^,^^4^ This surgical procedure is associated with significant morbidity, complications occur in 59% of cases, with pulmonary complications being the most common and anastomotic leakage the most severe.^5^

In 2015, an innovative surgical technique for the treatment of esophageal cancer, the Minimally Invasive transCervical oEsophagectomy (MICE), was developed in Japan. The technique is performed through a left collar incision, which gives access to the left recurrent laryngeal nerve; the nerve is localized, checked with intraoperative neuromonitoring, and kept intact. Once the cervical esophagus has been freed and cervical lymph node station (101L) has been dissected, the surgeon introduces a single port platform and insufflates the mediastinum, so that the avascular plane surrounding the mesoesophagus can be opened and extended downward through a combination of sharp and pneumatic dissection. The upper and mid esophagus, together with the associated lymph nodes, are thereby removed as a single specimen, with sparing of both recurrent laryngeal nerves, the thoracic duct, the azygos vein, both pleural surfaces, and the proximal portions of both vagal nerves. A wide upper mediastinal nodal clearance is carried out during this cervical phase, covering the bilateral paraesophageal (105, 108, and 110), paratracheal (106, 106tbR, and 106tbL), subcarinal (107), and bilateral recurrent laryngeal nerve (106recL and 106recR) stations. A laparoscopic transhiatal route is then used to connect the cervical and mediastinal fields. Gastric mobilization and abdominal nodal dissection follow (including stations 1, 2, 3, 7, 8a, 8p, 9, and 11), after which a gastric conduit is constructed, advanced to the neck, and joined to the esophagus with a handsewn end to side anastomosis.^6–8^

By maintaining pleural integrity, MICE may reduce postoperative pulmonary complications such as pneumonia, while facilitating oncological radicality. However, the extensive upper mediastinal dissection poses a risk of injury to the left recurrent laryngeal nerve, which may lead to (transient) vocal cord dysfunction and, consequently, aspiration and dysphonia.^8^^,^^9^

In 2019, MICE was introduced in Radboud University Medical Center in The Netherlands according to the IDEAL framework.^10^ While previous studies from Japan have compared MICE and TMIE, the applicability of these techniques in European populations remains unclear. Differences in tumor histology, patient demographics, and healthcare systems between Asia and Europe highlight the need for further investigation to determine whether the benefits observed in Japanese studies translate to this population.^11–13^

This study aims to compare the postoperative outcomes following MICE with standard care (TMIE) within a single-center setting. We hypothesize MICE may reduce postoperative pulmonary complications and severity of anastomotic leakage due to preservation of the pleurae.

## METHODS

### Study design

This study is a retrospective cohort study conducted at Radboud university medical center (Radboudumc) in Nijmegen, The Netherlands. Data were obtained from the Electronic Health Records (EHR). The study complies with a waiver (2025-17971) obtained from Medical Research Ethics Committees Eastern Netherlands (METC Oost-Nederland). As it involves only retrospective data collection from the EHR, it was not subject to the Dutch Medical Research Involving Human Subjects Act.

### Patient selection

We retrospectively reviewed the records of patients (≥18 years) diagnosed with resectable esophageal or gastro-esophageal junction carcinoma (cT1–4a N0–3M0) who underwent either MICE or TMIE at Radboudumc between January 2020 and December 2024. At our institution, Ivor Lewis TMIE is the standard surgical approach for distal esophageal and gastro–esophageal junction tumors. This comparator was selected because the ICAN trial demonstrated that an intrathoracic anastomosis after transthoracic MIE is associated with lower rates of anastomotic leakage, fewer severe postoperative complications, and a lower incidence of recurrent laryngeal nerve palsy compared with a cervical anastomosis.^14^ Patients were identified from the EHR. During the initial year of the MICE program, the indication for MICE was restricted to patients with high lymph node involvement, high tumors or squamous cell carcinoma. From 2021 onwards, the indication was expanded to include patients with distal esophageal adenocarcinoma, primarily based on shared decision making and patient preference. Patients were excluded if they underwent esophageal surgery other than MICE, TMIE (Ivor Lewis or McKeown); if they did not receive neoadjuvant treatment; if they underwent salvage surgery; if no resection was performed; or if an anastomosis was not created or the procedure was aborted because of extensive disease progression.

### Definitions

Postoperative complications were defined according to the oEsophageal Complications Consensus Group (ECCG) criteria and were recorded if they occurred within 30 days postoperatively or during the index admission.^15^ The severity of these complications was graded using the Clavien–Dindo classification and the overall burden of complications was calculated with the comprehensive complication index (CCI).^16^^,^^17^

Clinically relevant recurrent laryngeal nerve palsy (RLNP) was defined as dysphonia requiring vocal cord augmentation. All MICE patients were scheduled for postoperative assessment by a speech therapist and an otorhinolaryngologist. This evaluation included assessment of the presence and severity of dysphonia as well as a laryngoscopic examination to evaluate vocal cord mobility. In case of persistent vocal cord paralysis at 6–8 weeks postoperative, with a concomitant request for treatment by the patient, augmentation was directly performed under local anesthesia. Thyroplasty was performed in cases of vocal cord paralysis that persisted for more than one year. In the TMIE group, this dysphonia assessment including laryngoscopy was not routinely performed and was only carried out when clinically indicated.

### Outcomes

Demographic and baseline characteristics including pulmonary comorbidities defined by the international classification of diseases and related health problems (ICD-10),^18^ health status (ASA classification), smoking history, preoperative TNM-stage,^19^ neoadjuvant therapy (radio-, chemo-, or chemoradiotherapy), involvement of high lymph nodes, sex, and body mass index (BMI), were recorded to ensure representativeness. The primary outcome was the incidence of postoperative complications. Secondary outcomes included perioperative outcomes (duration of surgical procedure, blood loss, and conversion top open surgery), pulmonary complications comprising the individual underlying disorders (e.g. pneumonia, pleural effusion and pneumothorax), recurrent laryngeal nerve palsy (RLNP), hospital (re) admission, ICU stay, and morbidity. Overall morbidity was expressed using the CCI, which quantifies the cumulative severity of all postoperative complications on a scale from 0 (no complication) to 100 (death).

### Statistical analysis

Baseline patient characteristics in the unadjusted cohort were summarized as absolute numbers with percentages for categorical variables and as means with standard deviations for continuous variables. Inverse probability of treatment weighting (IPTW) was performed to minimize confounding between the MICE and TMIE groups. Propensity scores were estimated using multivariable logistic regression including the following covariates: ECOG performance status, sex, BMI, ASA classification, smoking history (never, former, or current), tumor location (proximal-mid or distal), clinical T stage (T1–T4), clinical N stage (N0–N3), involvement of high lymph nodes, pulmonary comorbidity, and neoadjuvant treatment (chemotherapy or chemoradiotherapy). Weights were calculated for the average treatment effect and extreme weights were trimmed at the 99th percentile to improve stability of the weighted estimates. Balance between treatment groups before and after weighting was evaluated using standardized mean differences (SMDs), with a SMD < 0.1 considered to indicate adequate balance.

After inverse probability of treatment weighting, categorical outcomes were reported as proportions with risk differences and 95% confidence intervals, estimated using generalized linear models with an identity link. Continuous outcomes were analyzed using linear regression models to obtain group means and mean differences with 95% confidence intervals. Standard errors were calculated using a robust variance estimator to account for weighting. In addition, as a sensitivity analysis, we performed a double robust analysis combining IPTW with outcome regression (i.e., augmented IPTW). This approach provides additional protection against model misspecification, because it remains valid if either the treatment model or the outcome model is correctly specified.^20^

The CCI was calculated for each patient based on all recorded Clavien–Dindo graded complications and was analyzed as a continuous outcome in the weighted cohort to reflect the overall postoperative complication burden.

Within the MICE cohort, additional unadjusted analyses were performed to assess the association between clinically relevant RLNP and postoperative pneumonia, and between pleural integrity and postoperative pneumonia. Absolute risks and RDs with 95% CI were calculated, and unadjusted associations were evaluated using Fisher exact tests. No adjusted analyses were performed because of the low event incidence.

All analyses were conducted using R (version 4.4.1, R Foundation for Statistical Computing, Vienna, Austria).

## RESULTS

**Fig. 1:**
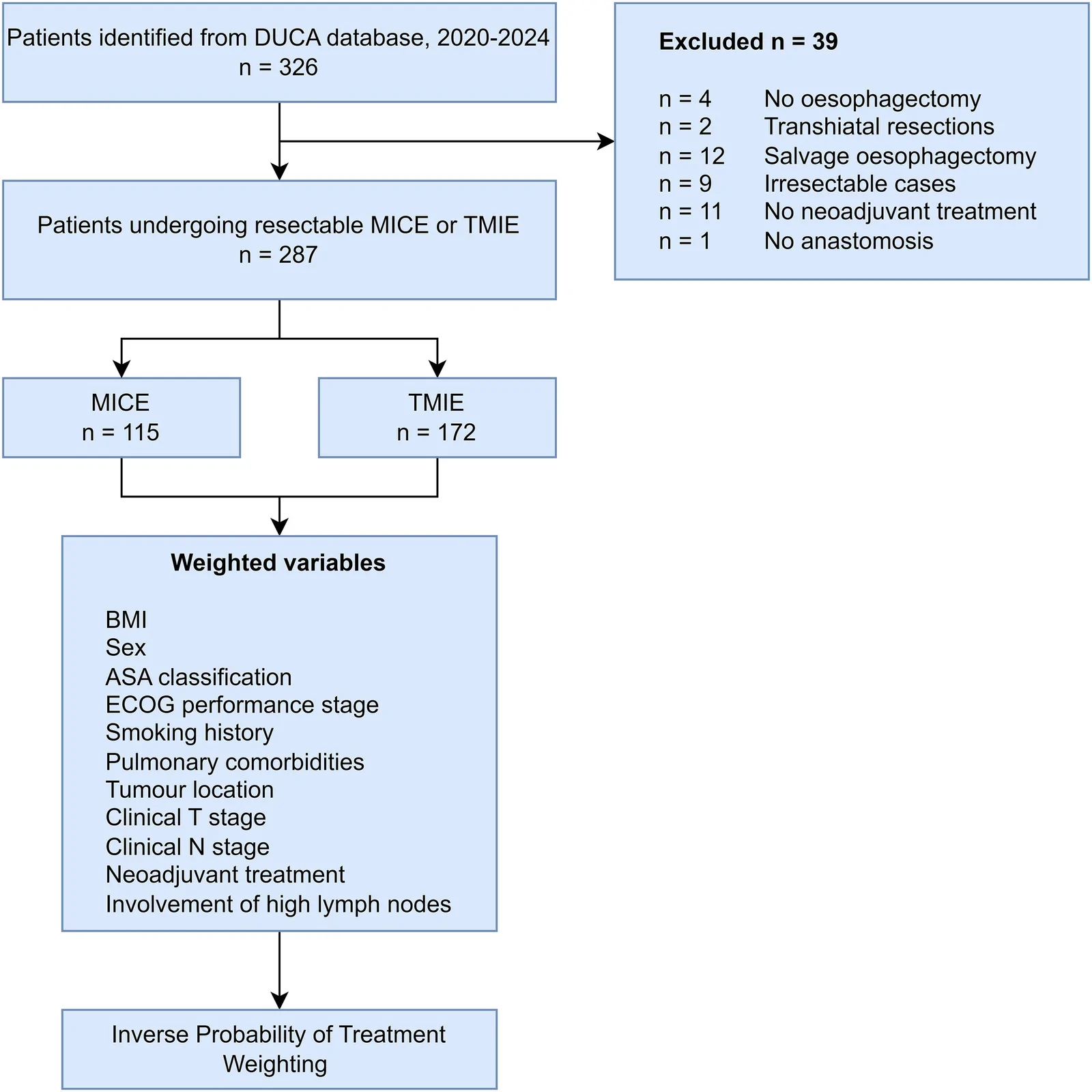
Flowchart of the study. ASA, American Society of Anaesthesiologists physical status classification system; BMI, body mass index; ECOG, Eastern Cooperative Oncology Group performance status; MICE, Minimally Invasive Cervical oEsophagectomy; MIE, Transthoracic Minimally Invasive oEsophagectomy.

In total, 326 patients were identified from the EHR who underwent esophageal surgery. Four patients were excluded as they did not undergo esophagectomy, two patients underwent a transhiatal esophagectomy, and in one case no anastomosis was made. A total of 12 patients underwent salvage procedures, and in nine cases no resection was performed due to intraoperative findings. Additionally, 11 patients had not received neoadjuvant treatment and were therefore excluded. This resulted in 287 patients being included in the study: 115 underwent MICE and 172 underwent TMIE (including 8 patients with cervical anastomosis). Figure 1 shows the flow chart of the study.

Table 1 presents the baseline characteristics of both groups. After applying inverse probability of treatment weighting all prespecified covariates were adequately balanced between the TMIE and MICE groups, except neoadjuvant therapy, its distribution differed substantially between the two groups and this imbalance could not be fully corrected by the weighting model. Residual imbalance in neoadjuvant therapy persisted after weighting and no additional adjustment for this variable was performed in the weighted outcome analyses. Table 2 shows the balance of baseline covariates between the TMIE and MICE groups before and after inverse probability of treatment weighting.

**Table 1:** Baseline patient characteristics of MICE and TMIE groups from the unadjusted cohort

Parameter	TMIE (*n* = 172)	MICE (*n* = 115)
**Age** (years (SD))	66.3 (8.5)	67.2 (7.8)
**Men**	123 (70.3)	86 (76.8)
**BMI** (mean (SD))	26.45 (4.66)	25.10 (4.11)
**ASA score**		
ASA 1	3 (1.7)	4 (3.6)
ASA 2	110 (62.9)	69 (61.6)
ASA 3	61 (34.9)	39 (34.8)
ASA 4	1 (0.6)	0 (0.0)
**ECOG performance status**		
0	117 (66.9)	92 (82.1)
1	57 (32.6)	20 (17.9)
2	1 (0.6)	0 (0.0)
**Smoking status**		
Never	48 (27.4)	24 (21.4)
Former	101 (57.7)	75 (67.0)
Current	26 (14.9)	13 (11.6)
**Pulmonary comorbidities** ^*^	32 (18.3)	17 (15.2)
**Tumor location**		
Proximal-mid	8 (4.6)	25 (22.3)
Distal	167 (95.4)	87 (77.7)
**T stage**		
T1	3 (1.7)	1 (0.9)
T2	27 (15.4)	24 (21.4)
T3	137 (78.3)	85 (75.9)
T4	8 (4.6)	2 (1.8)
**N stage**		
N0	45 (25.7)	32 (28.6)
N1	78 (44.6)	34 (30.4)
N2	48 (27.4)	39 (34.8)
N3	4 (2.3)	7 (6.2)
**Involvement of high lymph nodes** ^**†**^	8 (4.6)	28 (25.0)
**Tumor type**		
AC	153 (87.4)	91 (81.2)
SCC	18 (10.3)	19 (17.0)
Rest group	4 (2.3)	2 (1.8)
**Neoadjuvant therapy**		
Chemotherapy	48 (27.4)	1 (0.9)
Chemoradiotherapy	127 (72.6)	111 (99.1)
**Conversion to TMIE**	NA	8 (7.0)
**Conversion to open**	5 (2.9)	4 (3.5)

Values are *n* (%) unless otherwise indicated.

^*^Defined by the international classification of diseases and related health problems (ICD-10).

^†^Defined as lymph node stations 1–8.

AC, adenocarcinoma; BMI, body mass index; MICE, Minimally Invasive transCervical oEsophagectomy; NA, not applicable; SCC, squamous cell carcinoma; SD, standard deviation; TMIE, Transthoracic Minimally Invasive oEsophagectomy.

**Table 2:** Balance before and after inverse probability of treatment weighting

	Before IPTW			After IPTW		
Parameter	TMIE	MICE	SMD	TMIE	MICE	SMD
**Men**	69.8	77.4	−0.076	73.7	74.0	−0.003
**BMI** mean (SD)	26.44 (4.67)	25.15 (4.12)	−0.294	26.17 (4.34)	25.96 (4.42)	−0.048
**ASA score**						
ASA 1	1.7	3.5	0.017	1.8	2.1	0.003
ASA 2	62.8	61.7	−0.011	63.4	64.4	0.010
ASA 3	34.9	34.8	−0.001	34.4	33.5	−0.009
ASA 4	0.6	0.0	−0.006	0.4	0.0	−0.004
**ECOG performance status**						
0	66.3	82.6	0.163	72.6	77.4	0.048
1	33.1	17.4	−0.158	27.1	22.6	−0.045
2	0.6	0.0	−0.006	0.4	0.0	−0.004
**Smoking status**						
Never	27.9	20.9	−0.070	25.5	24.2	−0.013
Former	57.0	67.8	0.109	61.9	64.6	0.027
Current	15.1	11.3	−0.038	12.6	11.2	−0.014
**Pulmonary comorbidities** ^*^	18.6	14.8	−0.038	17.0	15.6	−0.014
**Tumor location**			−0.171			−0.046
Proximal-mid	4.7	21.7		08.9	13.5	
Distal	95.3	78.3		91.1	86.5	
**T stage**						
T1	1.7	0.9	−0.009	1.5	0.9	−0.005
T2	15.7	20.9	0.052	18.4	21.4	0.030
T3	77.9	76.5	−0.014	76.5	75.5	−0.010
T4	4.7	1.7	−0.029	3.6	2.2	−0.014
**N stage**						
N0	26.2	27.8	0.017	26.7	30.9	0.042
N1	44.2	31.3	−0.129	41.6	38.5	−0.031
N2	27.3	34.8	0.075	27.6	26.5	−0.011
N3	2.3	6.1	0.038	4.0	4.1	0.001
**Involvement of high lymph nodes** ^**†**^	4.7	24.3	0.197	10.2	15.0	0.048
**Neoadjuvant therapy**			0.270			0.152
Chemotherapy	27.9	0.9		17.6	2.4	
Chemoradiotherapy	72.1	99.1		82.4	97.6	

Values are in % unless otherwise indicated.

^*^Defined by the international classification of diseases and related health problems (ICD-10).

^**†**^Defined as lymph node stations 1–8.

BMI, body mass index; ECOG, Eastern Cooperative Oncology Group; IPTW, inverse probability of treatment weighting; MICE, Minimally Invasive transCervical oEsophagectomy; SMD, standard mean difference; TMIE, Transthoracic Minimally Invasive oEsophagectomy.

### Postoperative complications

An overview of postoperative complications (within 30 days or during the index admission) in the MICE and TMIE groups is presented in Table 3. In the unadjusted cohort the overall complications were more frequent after MICE than TMIE (63.5% vs. 45.3%, RD: 18.1, 95% CI: 6.6–29.7), and remained after IPTW (60.3% vs. 43.9%, RD: 16.4, 95% CI: 3.3–29.4). Sensitivity analysis using double robust analysis showed similar results (63.5% vs. 45.3%, RD: 16.8, 95% CI: 4.4–29.2). The results of the double robust analysis for the individual postoperative complication groups and for the perioperative outcomes are presented in Tables S3 and S4, respectively.

**Table 3:** Incidence of postoperative complications and proportion of Clavien Dindo 3b or higher classified complications among patients with the specific complication in MICE and TMIE after inverse probability of treatment weighting

Parameter	TMIE	MICE	RD/MD (95% CI)
**Complications** ^*^			
**Occurrence of any complication**	43.9	60.3	16.4 (3.3–29.4)
Clavien-Dindo ≥3b	23.9	21.4	−2.5 (−18.4 to 23.0)
**Occurrence of any complication without RLNP**	43.4	48.8	5.4 (−31.1 to 23.1)
Clavien-Dindo ≥3b	24.2	26.5	2.2 (−21.6 to 23.5)
**Pulmonary**	21.8	18.9	−2.9 (−17.1 to 12.5)
Clavien-Dindo ≥3b	29.6	22.5	−7.1 (−36.1 to 28.9)
Pneumonia	10.7	15.5	4.8 (−7.4 to 17.9)
Clavien-Dindo ≥3b	12.6	14.2	1.6 (−28.8 to 36.8)
**Gastro-intestinal**	14.6	11.9	−2.7 (−14.8 to 10.1)
Clavien-Dindo ≥3b	36.9	52.1	15.2 (−30.6 to 60.0)
Anastomotic leakage^*^	8.1	5.6	−2.4 (−11.5 to 7.3)
Clavien-Dindo ≥3b	43.6	58.8	15.2 (−35.6 to 66.0)
**Neurological/psychiatric**	3.6	35.0	31.3 (18.0 to 43.6)
Clavien-Dindo ≥3b	0.0	0.0	0.0 (−39.0 to 9.9)
Clinically relevant RLNP	0.0	30.4	30.4 (19.7 to 40.8)
Augmentation	NA	79.0	NA (NA)
Augmentation followed by thyroplasty	NA	21.0	NA (NA)
Clavien-Dindo ≥3b	NA	0.0	NA (NA)
**Cardiac**	15.3	21.1	5.8 (−8.1 to 20.3)
Clavien-Dindo ≥3b	4.7	11.4	6.7 (−15.8 to 32.1)
**Urological**	3.6	8.4	4.8 (−3.9 to 14.3)
Clavien-Dindo ≥3b	0.0	0.0	0.0 (−49.0 to 29.9)
**Thromboembolic**	4.5	1.7	−2.8 (−8.6 to 5.4)
Clavien-Dindo ≥3b	0.0	0.0	0.0 (−39.0 to 65.8)
**Infections**	9.6	9.2	−0.4 (−10.4 to 11.6)
Clavien-Dindo ≥3b	0.0	7.4	7.4 (−19.3 to 47.1)
**Wound/diaphragm**	0.9	4.0	3.0 (−2.4 to 10.3)
Clavien-Dindo ≥3b	0.0	50.6	50.6 (−64.3 to 85.0)
**Chyle leak**	5.6	2.6	−3.0 (−10.5 to 4.4)
Clavien-Dindo ≥3b	8.1	45.9	37.8 (−28.4 to 85.0)
**Other**	6.2	6.1	−0.1 (−8.1 to 10.2)
Clavien-Dindo ≥3b	0.0	19.3	19.3 (−22.3 to 62.4)
**CCI whole groups patients (mean)**	15.78	25.32	9.54 (4.13 to 14.94)
**CCI whole groups patients, without RLNP (mean)**	15.78	22.12	6.33 (1.14 to 11.53)

Values are in % unless otherwise indicated. Clavien–Dindo ≥3b percentages are calculated within each complication category (i.e. proportion of cases of that complication meeting ≥3b criteria).

^*^Defined by the Esophagectomy Complications Consensus Group.

CCI, comprehensive complication index; CI, confidence interval; MD, mean difference; MICE, Minimally Invasive transCervical oEsophagectomy; NA, not applicable; NE, not estimated; RD, Risk Difference; RLNP, recurrent laryngeal nerve palsy; TMIE, Transthoracic Minimally Invasive oEsophagectomy.

This difference in overall postoperative complication rate between MICE and TMIE was largely attributable to the occurrence of recurrent laryngeal nerve palsy (RLNP) (30.4% vs. 0.0%). When RLNP was excluded from the overall postoperative complication rate, the difference decreased to 48.8% vs. 43.4% (RD: 5.4%, 95% CI: −13.1 to 23.1). No statistically significant difference was found in the incidence of anastomotic leakage between the two groups (5.6% vs. 8.1%, RD: −2.4; 95% CI: –11.5 to 7.3).

The incidence of postoperative pulmonary complications was comparable between the weighted groups (MICE 18.9% vs. TMIE 21.8%; RD: –2.9, 95% CI: −17.1 to 12.5). Pneumonia was the most common pulmonary complication, occurring in 15.5% of MICE and 10.7% of TMIE and patients (RD: 4.8, 95% CI: −7.4 to 17.9). Other pulmonary complications, including pleural effusion, pneumothorax, atelectasis, respiratory failure, acute aspiration, acute respiratory distress syndrome, tracheobronchial injury, and prolonged chest tube drainage for more than 10 days, were infrequent and showed no statistically significant difference between groups (supplementary information, Table S2).

New onset or worsening of dysphonia occurred in 81.3% of the MICE patients. In the majority of these patients, dysphonia was transient and resolved without intervention (61.4%). However, 38.6% of patients with dysphonia required vocal cord augmentation, reflecting clinically relevant RLNP.

When expressed relative to the entire MICE cohort, clinically relevant RLNP was observed in 30.4% of patients. Of patients with clinically relevant RLNP, 79.0% underwent augmentation at 6–8 weeks postoperative, while 21.0% required both augmentation and subsequent thyroplasty due to persisting vocal cord paralysis after one year.

Other types of complication subgroups (e.g. including cardiac, gastrointestinal, urological, thromboembolic, infectious, wound, chyle leak, and other complications) showed no statistically significant differences between MICE and TMIE (supplementary information, Table S1).

### Severity of postoperative complications

After IPTW, the CCI was significantly higher in the MICE group (25.32 vs. 15.78; MD: 9.54; 95% CI: 4.13–14.94). This difference was primarily driven by the high frequence of clinically relevant RLNP (requiring treatment), classified within the neurological/psychiatric category. Although statistically significant, this mean difference of 9.54 points may not be clinically meaningful. A recent anchor based analysis proposed a minimal important difference of 12 CCI points to reflect a patient relevant difference.^21^

Among all postoperative complications, 21.4% of the patients in the MICE group had severe complications (Clavien–Dindo grade >3b), compared to 23.9% of the TMIE group. An overview of the severity of postoperative complications per complication subgroup is shown in Table 3. The corresponding crude, unweighted results are provided in Table S1.

### Perioperative outcomes and (re)admission

The mean duration of the procedure (454.8 vs. 325.4; MD: 129.4; 95% CI: 108.0–150.8) and operative time (365.8 vs. 253.7; MD: 112.1; 95% CI: 92.4–131.8) was significantly higher in the MICE group. Although the MICE technique allows for an extensive upper mediastinal lymph node dissection, in which the paratracheal lymph node stations are systematically resected, the number of resected lymph nodes was higher, but not statistically significantly different between the two groups (31.1 vs. 27.4, RD: 3.6, 95% CI: 1.4–5.9). This higher lymph node yield was primarily driven by dissection of the high intrathoracic stations, namely the paratracheal, recurrent laryngeal nerve, and aorto-pulmonary window nodes (stations 106, 106recR, 106recL, and 106tbL), which are dissected during the transcervical phase in MICE and during the thoracic phase in a McKeown MIE. These were resected in 96.3% of MICE patients compared with 19.3% of MIE patients (RD: 77.1, 95% CI: 68.5–85.6). The subcarinal station (107), likewise approached during the transcervical phase in MICE and the thoracic phase in MIE, was also resected slightly more frequently in the MICE group (RD: 3.0, 95% CI: 0.3–5.7). Dissection of the lower mediastinal and abdominal stations is performed laparoscopically in both techniques, and this resulted in comparable dissection rates between MICE and MIE, with no significant differences for the lower paraesophageal stations (108 and 110) or the N1 and N2 abdominal stations (Table 4).^22^^,^^23^

**Table 4:** Comparison of perioperative outcomes and (re)admission rates between MICE and MIE after inverse probability of treatment weighting

Parameter	TMIE	MICE	RD/MD (95% CI)
**Perioperative outcomes**			
Duration of procedure (min), mean	325.4	454.8	129.4 (108.0 to 150.8)
Operative time (min), mean	253.7	365.8	112.1 (92.4 to 131.8)
Blood loss (mL), mean	139.1	200.5	61.4 (−100.9 to 223.8)
Conversion to open (%)	2.5	5.0	2.5 (−3.9 to 11.1)
**Oncological outcomes**			
Resected lymph nodes, mean	27.4	31.1	3.6 (1.4 to 5.9)
Intrathoracic—high Paratracheal, along the recurrent laryngeal nerve and aorto-pulmonary window (106, 106recR, 106recL, and 106tbL)	19.3	96.3	77.1 (68.5 to 85.6)
Intrathoracic—low Subcarinal (107)	97.0	100.0	3.0 (0.3 to 5.7)
Paraesophageal (108, 110)	99.6	100.0	0.4 (−0.4 to 1.2)
N1 lymph node stations of the stomach (at least 3 of 6 node stations (1, 2, 3, 7, 8a, 8p, 9, and 11))	99.5	100.0	0.5 (−0.5 to 1.5)
N2 lymph node stations of the stomach (at least 3 of 6 node stations)	98.4	96.5	−1.9 (−6.5 to 2.7)
Distant lymph node stations	5.2	7.7	2.5 (−3.6 to 8.6)
Other (e.g. cervical)	0.4	4.5	4.0 (0.1 to 8.0)
Positive lymph nodes (*n*), mean	2.1	2.0	−0.1 (−0.9 to 0.7)
Radicality (%)			
R0 resection	97.2	97.6	0.4 (−6.3 to 6.7)
R1 resection	2.4	1.8	−0.6 (−5.7 to 6.7)
Not assessable	0.4	0.7	0.3 (−3.8 to 5.7)
**(Re)admission**			
LOS, hospital (days), mean	11.8	12.4	0.6 (−2.4 to 3.7)
LOS, ICU (days), mean	2.2	2.8	0.6 (−1.2 to 2.3)
Readmission within 30 days (%)	13.3	18.6	5.3 (−8.2 to 18.6)

CI, confidence interval; ICU, intensive care unit; ; LOS, length of stay; MD, mean difference; MICE, minimally invasive transCervical oEsophagectomy; RD, risk difference; TMIE, Transthoracic Minimally Invasive oEsophagectomy. Lymph node stations were classified according to the Japanese Classification of Esophageal Cancer (11th Edition).

No statistically significant difference was observed in mean blood loss, conversion rate, R0 resection rates, length of (ICU)stay or readmission. An overview of perioperative outcomes and (re)admission rates comparing MICE and TMIE is presented in Table 4.

### Association of recurrent laryngeal nerve palsy and pleural integrity with pneumonia

An analysis was performed in the unweighted group of MICE patients to evaluate the association between the absence versus the presence of clinically relevant RLNP and postoperative pneumonia. No statistically significant association was observed, postoperative pneumonia occurred in 12.2% of patients without clinically relevant RLNP and in 23.7% of those with clinically relevant RLNP (RD: 11.5%, 95% CI: −3.9% to 27.0%).

Pleural integrity was breached in 69.6% of the MICE patients. There was no significant difference in incidence of postoperative pneumonia between patients in whom the pleura was opened and those in whom pleural integrity was preserved (16.2% vs. 14.7%, RD: 1.5%, 95% CI: −12.8% to 15.9%). No adjusted analysis was performed because of the low event incidence and small sample size.

## DISCUSSION

This retrospective single center cohort study is the first comparison of postoperative complications between patients undergoing MICE and TMIE for esophageal cancer in a European setting. In this IPTW adjusted analysis, overall postoperative complications were observed more frequently after MICE. Although hypothesized that MICE would be superior to TMIE in terms of pulmonary complications, this IPTW analysis showed similar pulmonary outcomes for MICE and TMIE. Also, for other important postoperative complications (i.e. anastomotic leakage) no significant differences could be demonstrated between MICE and TMIE, with the exception of a high rate of RLNP in the MICE group, which is inevitably associated with the transcervical approach.

When complication categories, such as cardiac, urological, gastrointestinal, thromboembolic, and infectious complications, were analyzed, no relevant differences were observed between groups. In addition, no statistically significant differences were observed in the incidence of pneumonia or anastomotic leakage. Pleural breach occurred in 69.6% of patients undergoing MICE. However, this percentage includes both intentional and mostly small and unintentional pleural defects and should therefore not be interpreted as equivalent to a transthoracic approach. Importantly, even when pleural entry occurs during MICE, the procedure does not require single-lung ventilation or intentional collapse of the lung with positive pressure to establish the operative field, as is required during a transthoracic approach. Therefore, pleural breach during MICE does not necessarily imply complete loss of the potential benefits associated with avoiding transthoracic access. As these data include the initial implementation phase of MICE at our institution, the observed rate of pleural breach may partly reflect the learning curve associated with adoption of the technique. Despite the cervical anastomosis in the MICE group, the anastomotic leakage rate of 5.6% was relatively low compared with the 21.9% reported for cervical anastomoses in the Dutch Upper Gastrointestinal Cancer Audit.^24^

This may be due to the optimal exposure provided after cervical lymph node resection and lower level of esophago-gastric anastomosis, which actually results in a high intrathoracic anastomosis instead of a true cervical one in McKeown esophagectomy.

The large difference in RLNP rates between the MICE (30.4%) and TMIE (0%) group in this study are attributable to institutional practice patterns, specifically the routine performance of Ivor Lewis transthoracic minimally invasive esophagectomies at our center. In contrast to the McKeown and MICE techniques, this approach does not include an extensive upper mediastinal (paratracheal) lymph node dissection and thereby omits the risk of RLNP. This choice is supported by the ICAN trial, which demonstrated that an intrathoracic anastomosis after transthoracic MIE is associated with a lower rate of anastomotic leakage, fewer severe complications, and a lower incidence of recurrent laryngeal nerve palsy than a cervical anastomosis.^25^

Although RLNP occurred frequently after MICE, it appears to be a largely manageable; its clinical relevance and acceptability warrants further investigation. Symptoms are transient in most cases and can usually be effectively managed with our speech therapy care pathway and vocal cord augmentation, whereas approximately one fifth of patients require definitive surgical intervention, such as thyroplasty.

Furthermore, no significant association was observed between RLNP and the occurrence of postoperative pneumonia. Although RLNP after esophagectomy is a well-known risk factor for pulmonary complications due to aspiration associated with impaired vocal cord mobility, our findings indicate that RLNP (transient or requiring treatment) does not translate into a significantly higher risk of postoperative pneumonia.^26^

These results suggest that compensatory mechanisms and our early postoperative management strategies, such as early consultation of a speech therapist and routine endoscopic evaluation of swallowing (FEES), may mitigate the potential pulmonary risks of vocal cord dysfunction. While most RLNP resolve without treatment, clinicians should remain attentive to the short-term management of associated symptoms. Interventions, such as augmentation in an early stage, can significantly reduce these symptoms. As surgical experience with MICE grows and RLN protective measures are adopted (continuous nerve monitoring and lateralization techniques), it is likely that RLNP rates will decrease.

A key strength of this study is its comprehensive and systematically collected dataset, encompassing detailed perioperative variables and postoperative outcomes. The use of inversed probability of treatment weighting minimized confounding and enabled a balanced comparison between MICE and TMIE.

Several limitations of this study should be acknowledged. First, although inverse probability of treatment weighting achieved adequate balance for all prespecified covariates except one, the distribution of neoadjuvant therapy remained substantially different and could not be fully corrected by the weighting model. Nearly, all patients in the MICE group received neoadjuvant chemoradiotherapy, whereas a substantial proportion of TMIE patients received neoadjuvant chemotherapy. As neoadjuvant treatment may influence tissue characteristics, operative complexity, and postoperative recovery, residual confounding related to treatment strategy cannot be excluded. However, to further address this residual imbalance, a doubly robust analysis was performed in which neoadjuvant treatment was included in the outcome model in addition to its inclusion in the propensity score model. This analysis did not meaningfully alter the effect estimates compared with the primary IPTW analysis, that additionally accounted for neoadjuvant chemoradiotherapy did not meaningfully alter the effect estimates, indicating that imbalance in neoadjuvant treatment is unlikely to fully explain the observed differences. Nevertheless, residual confounding cannot be entirely excluded, and the findings should therefore be interpreted with appropriate caution.

Second, interpretation of RLNP rates should be undertaken with caution. The large difference in RLNP incidence between groups is primarily explained by institutional practice patterns, as TMIE at our center is routinely performed as an Ivor Lewis procedure without extensive upper mediastinal lymph node dissection, thereby largely avoiding the risk of RLNP. However, postoperative surveillance also differed between groups. Patients undergoing MICE routinely underwent speech therapy assessment and laryngoscopy, whereas TMIE patients were only evaluated when clinically indicated. To reduce the risk of overestimating RLNP in the MICE group due to more intensive surveillance, the outcome was defined as clinically relevant RLNP requiring intervention. Nevertheless, mild or asymptomatic RLNP may have been under detected in the TMIE group, and some degree of detection bias cannot be excluded.

Third, the higher incidence of postoperative complications may partly reflect differences in procedural maturity between TMIE and MICE. As this cohort included patients from the start of the MICE program, outcomes may have been influenced by the learning phase, which is typically associated with longer operative times, greater technical variability, and learning associated morbidity.^27^^,^^28^ Consistent with this, the incidence of recurrent laryngeal nerve palsy decreased over the study period, an observation in keeping with a learning-curve effect. Because of the retrospective design and limited sample size, however, it was not possible to determine to what extent the observed morbidity is attributable to the intrinsic characteristics of the MICE procedure versus the learning curve associated with its adoption. Staffing changes during the study period further resulted in overlapping individual learning curves, precluding a meaningful temporal analysis of overall morbidity. Future studies evaluating outcomes after maturation of the learning curve are required to better define the safety and efficacy of MICE.

These findings also bear on how MICE should be positioned among the available surgical approaches. The technique may be regarded both as a potentially pleura sparing, minimally invasive alternative that avoids transthoracic access with the theoretical potential to reduce thoracic surgical trauma and pulmonary morbidity, and as a procedure that permits a systematic upper mediastinal lymphadenectomy comparable in extent to a McKeown dissection but without thoracic access. In addition to its potential pulmonary benefits, a cervical anastomosis may reduce the clinical consequences of anastomotic leakage, as leaks are generally contained in the neck and can be drained more readily than intrathoracic leaks. The relevance of each of these aspects is likely to differ by tumor type. Our cohort consisted predominantly of distal adenocarcinomas. For upper and mid thoracic carcinomas, which are underrepresented in the present cohort but carry a higher propensity for nodal metastases to the upper mediastinal and recurrent laryngeal nerve stations, the more extensive nodal clearance afforded by MICE may theoretically be of greater value. Whether the more extensive upper mediastinal lymphadenectomy achievable with MICE translates into an oncological benefit remains uncertain. In particular, the clinical and oncological relevance of the observed differences in lymph node retrieval cannot be determined from the present study, and this question, together with the optimal tumor indications for MICE, should be addressed in future comparative studies.

Future research should focus on long-term outcomes, including overall and disease-free survival, to provide a more comprehensive understanding of the benefits and risks associated with MICE. In addition, clear indications and selection criteria for MICE need to be defined, as current guidance remains limited and may influence both outcomes and comparability across centers. Ultimately, a multicenter randomized controlled trial with clearly defined indications is needed to validate the generalizability of our findings and provide high-level evidence on the comparative safety and efficacy of MICE versus TMIE.

In conclusion, this study demonstrates that overall postoperative complication rates were higher after MICE, this difference was mainly attributable to a higher incidence of transient RLNP, which is inevitably associated with the transcervical approach. However, rates of pneumonia and anastomotic leakage were similar between the two techniques. As these data include cases performed during the early experience with MICE, complication rates may improve as experience accumulates across surgical teams. These findings highlight the need for further evaluation and optimization of the MICE procedure prior to broader implementation.

## Supplementary Material

supplementary_material_doag106

## Data Availability

Data is available on request.

## Notes

Justin G. A. Grootenhuis and Sam Wijers contributed equally to this work.
