# Supplementary material for: Postoperative complications after Minimally Invasive transCervical oEsophagectomy (MICE) versus Transthoracic Minimally Invasive oEsophagectomy (TMIE): an inverse probability of treatment weighted analysis
Source: Dis Esophagus. 2026 Sep 22;39(5):doag106. doi: 10.1093/dote/doag106 (PMC13596241; doi:10.1093/dote/doag106)
Supplement: Supplementary file 1 [file supplementary_material_doag106.docx]

**Supplementary Materials - Index**

| **Supplementary Figures and Tables** |  |
| --- | --- |
| Table S1 | *Page 2* |
| Table S2 | *Page 3* |
| Table S3 | *Page 4* |
| Table S4 | *Page 5* |

| **Table S1 Incidence of postoperative complications and incidence of Clavien–Dindo 3b or higher classified complications in patients undergoing MICE and TMIE after inverse probability weighting.** | | | | | | | |
| --- | --- | --- | --- | --- | --- | --- | --- |
|  |  | **Before IPTW** | | | **After IPTW** | | |
| **Parameter** | | **TMIE** | **MICE** | **RD / MD (95% CI)** | **TMIE** | **MICE** | **RD / MD (95% CI)** |
| **Complications^§^ (%)** | |  |  |  |  |  |  |
| **Occurrence of any complication** | | 45.3 | 63.5 | 18.1 (6.6 to 29.7) | 43.9 | 60.3 | 16.4 (3.3 to 29.4) |
| Clavien-Dindo ≥3b | | 23.1 | 21.9 | -1.2 (-19.6 to 17.6) | 23.9 | 21.4 | -2.5 (-18.4 to 23.0) |
| **Occurrence of any complication without RLNP** | | 44.8 | 51.3 | 6.5 (-10.0 to 22.7) | 43.4 | 48.8 | 5.4 (-13.1 to 23.1) |
| Clavien-Dindo ≥3b | | 23.4 | 27.1 | 3.7 (-16.5 to 24.3) | 24.2 | 26.5 | 2.2 (-21.6 to 23.5) |
|  | |  |  |  |  |  |  |
| **Pulmonary** | | 23.8 | 20.0 | -3.8 ( -17.0 to 10.1) | 21.8 | 18.9 | -2.9 (-17.1 to 12.5) |
| Clavien-Dindo ≥3b | | 26.8 | 26.1 | -0.7 (-29.4 to 30.8) | 29.6 | 22.5 | -7.1 (-36.1 to 28.9) |
| Pneumonia | | 12.2 | 15.7 | 3.4 (-7.8 to 15.3) | 10.7 | 15.5 | 4.8 (-7.4 to 17.9) |
| Clavien-Dindo ≥3b | | 9.5 | 16.7 | 7.1 (-23.1 to 36.6) | 12.6 | 14.2 | 1.6 (-28.8 to 36.8) |
| **Gastro-intestinal** | | 16.3 | 11.3 | -5.0 (-15.8 to 6.9) | 14.6 | 11.9 | -2.7 (-14.8 to 10.1) |
| Clavien-Dindo ≥3b | | 35.7 | 53.8 | 18.1 (-25.0 to 56.1) | 36.9 | 52.1 | 15.2 (-30.6 to 60.0) |
| Anastomotic leakage**^§^** | | 8.7 | 7.0 | -1.8 (-10.3 to 7.8) | 8.1 | 5.6 | -2.4 (-11.5 to 7.3) |
| Clavien-Dindo ≥3b | | 40.0 | 62.5 | 22.5 (-66.5 to 33.7) | 43.6 | 58.8 | 15.2 (-64.9 to 43.0) |
| **Neurological / psychiatric** | | 3.5 | 37.4 | 33.9 (21.7 to 44.9) | 3.6 | 35.0 | 31.3 (18.0 to 43.6) |
| Clavien-Dindo ≥3b | | 0.0 | 0.0 | 0.0 (-39.0 to 8.2) | 0.0 | 0.0 | 0.0 (-39.0 to 9.9) |
| Clinically relevant RLNP | | 0.0 | 33.0 | 33.0 (22.9 to 42.1) | 0.0 | 30.4 | 30.4 (19.7 to 40.8) |
| Augmentation | | NA | 81.8 |  | NA | 79.0 |  |
| Augmentation followed by thyroplasty | | NA | 19.4 |  | NA | 21.0 |  |
| Clavien-Dindo ≥3b | | NA | 0.0 |  | NA | 0.0 |  |
| **Cardiac** | | 16.9 | 20.9 | 4.0 (-8.7 to 17.2) | 15.3 | 21.1 | 5.8 (-8.1 to 20.3) |
| Clavien-Dindo ≥3b | | 3.4 | 4.2 | 0.7 (-16.4 to 19.6) | 4.7 | 11.4 | 6.7 (-15.8 to 32.1) |
| **Urological** | | 2.9 | 8.7 | 5.8 (-1.8 to 14.0) | 3.6 | 8.4 | 4.8 (-3.9 to 14.3) |
| Clavien-Dindo ≥3b | | 0.0 | 0.0 | 0.0 (-43.4 to 27.8) | 0.0 | 0.0 | 0.0 (-49.0 to 29.9) |
| **Thromboembolic** | | 4.1 | 1.7 | -2.3 (-7.7 to 4.1) | 4.5 | 1.7 | -2.8 (-8.6 to 5.4) |
| Clavien-Dindo ≥3b | | 0.0 | 0.0 | 0.0 (-35.4 to 65.8) | 0.0 | 0.0 | 0.0 (-39.0 to 65.8) |
| **Infections** | | 9.3 | 9.6 | 0.3 (-9.2 to 10.5) | 9.6 | 9.2 | -0.4 (-10.4 to 11.6) |
| Clavien-Dindo ≥3b | | 0.0 | 10.0 | 10.0 (-18.6 to 40.4) | 0.0 | 7.4 | 7.4 (-19.3 to 47.1) |
| **Wound / Diaphragm** | | 0.6 | 3.5 | 2.9 (-1.9 to 8.5) | 0.9 | 4.0 | 3.0 (-2.4 to 10.3) |
| Clavien-Dindo ≥3b | | 0.0 | 50.0 | 50.0 (-64.3 to 85.0) | 0.0 | 50.6 | 50.6 (-64.3 to 85.0) |
| **Chyle leak** | | 4.1 | 3.5 | -0.6 (-6.8 to 6.6) | 5.6 | 2.6 | -3.0 (-10.5 to 4.4) |
| Clavien-Dindo ≥3b | | 14.3 | 50.0 | 35.7 (-36.3 to 82.4) | 8.1 | 45.9 | 37.8 (-28.4 to 85.0) |
| **Other** | | 7.0 | 6.1 | -0.9 (-8.8 to 8.0) | 6.2 | 6.1 | -0.1 (-8.1 to 10.2) |
| Clavien-Dindo ≥3b | | 0.0 | 33.3 | 33.3 (-14.6 to 70.0) | 0.0 | 19.3 | 19.3 (-22.3 to 62.4) |

*Table S1 V*alues are in % unless otherwise indicated. *Clavien–Dindo ≥3b percentages are calculated within each complication category (i.e., proportion of cases of that complication meeting ≥3b criteria).* § *Defined by the Esophagectomy Complications Consensus Group.* IPTW, Inverse probability of treatment weighted *MICE, Minimally Invasive transCervical oEsophagectomy; TMIE, Transthoracic Minimally Invasive oEsophagectomy; RLNP, recurrent laryngeal nerve palsy; CCI, Comprehensive Complication Index; RD, Risk Difference; MD, Mean Difference; CI, Confidence Interval; NA, Not Applicable; NE, Not Estimated*

| ***Table S2 Pulmonary Outcomes unweighted and IPTW weighted with Risk Difference and 95 percent confidence interval*** | | | | | | |
| --- | --- | --- | --- | --- | --- | --- |
|  | **Before IPTW** | | | **After IPTW** | | |
| **Outcome** | **MIE** | **MICE** | **RD / MD (95% CI)** | **MIE** | **MICE** | **RD / MD (95% CI)** |
| Pneumonia | 12.2 | 15.7 | 3.4 (-7.8 to 15.3) | 10.7 | 15.5 | 4.8 (-7.4 to 17.9) |
| Pleural effusion requiring drainage | 8.7 | 4.3 | -4.4 (-12.0 to 4.4) | 6.8 | 3.5 | -3.3 (-10.9 to 5.5) |
| Pneumothorax requiring treatment | 3.5 | 0.0 | -3.5 (-7.4 to 1.6) | 3.6 | 0.0 | -3.6 (-8.3 to 2.4) |
| Atelectasis due to mucus plugging requiring bronchoscopy | 1.7 | 0.0 | -1.7 (-5.0 to 2.6) | 2.1 | 0.0 | -2.1 (-6.2 to 3.2) |
| Respiratory failure requiring reintubation | 1.7 | 3.5 | 1.7 (-3.6 to 8.0) | 2.1 | 2.8 | 0.7 (-5.1 to 8.2) |
| Acute aspiration | 0.6 | 0.9 | 0.3 (-3.1 to 4.7) | 0.6 | 1.9 | 1.3 (-3.4 to 7.3) |
| Acute respiratory distress syndrome | 1.7 | 0.9 | -0.9 (-4.8 to 4.2) | 1.3 | 0.8 | -0.5 (-5.0 to 5.4) |
| Tracheobronchial injury | 0.6 | 0.0 | -0.6 (-3.2 to 3.1) | 0.4 | 0.0 | -0.4 (-4.0 to 3.8) |
| Prolonged chest tube drainage for more than 10 days | 0.0 | 0.0 | 0.0 (-2.2 to 3.2) | 0.0 | 0.0 | 0.0 (-2.7 to 3.9) |
| Tracheostomy | 0.0 | 0.0 | 0.0 (-2.2 to 3.2) | 0.0 | 0.0 | 0.0 (-2.7 to 3.9) |

*Table S2 Values are in % unless otherwise indicated; IPTW, Inverse probability of treatment weighted; MICE, Minimally Invasive transCervical oEsophagectomy; TMIE, Transthoracic Minimally Invasive oEsophagectomy; RD, Risk Difference; MD, Mean Difference; CI, Confidence Interval;*

| **Table S3. Postoperative complications, doubly robust (AIPW, augmented inverse probability of treatment weighting) analysis.** | | | |
| --- | --- | --- | --- |
| **Parameter** | **TMIE** | **MICE** | **RD / MD (95% CI)** |
|  |  |  |  |
| **Complications^§^** |  |  |  |
| **Occurrence of any complication** | 45.3 | 63.5 | 16.8 (4.4 to 29.2) |
| Clavien-Dindo ≥3b | 10.5 | 15.7 | 8.2 (-3.2 to 19.7) |
| **Occurrence of any complication without RLNP** | 44.8 | 51.3 | 6.9 (-4.9 to 18.7) |
| Clavien-Dindo ≥3b | 10.5 | 15.7 | 7.7 (-3.4 to 18.8) |
|  |  |  |  |
| **Pulmonary** | 23.8 | 20.0 | -3.1 (-13.3 to 7.2) |
| Clavien-Dindo ≥3b | 6.4 | 5.2 | -1.4 (-6.6 to 3.9) |
| Pneumonia | 12.2 | 15.7 | 3.2 (-5.2 to 11.6) |
| Clavien-Dindo ≥3b | 1.2 | 2.6 | NE |
| **Gastro-intestinal** | 16.3 | 11.3 | -2.8 (-11.1 to 5.6) |
| Clavien-Dindo ≥3b | 5.8 | 6.1 | 2.7 (-5.7 to 11.0) |
| Anastomotic leakage**^§^** | 8.7 | 7.0 | -2.5 (-8.6 to 3.6) |
| Clavien-Dindo ≥3b | 3.5 | 4.3 | 0.3 (-4.2 to 4.8) |
| **Neurological / psychiatric** | 3.5 | 37.4 | 27.7 (18.9 to 36.5) |
| Clavien-Dindo ≥3b | 0.0 | 0.0 | NE |
| Clinically relevant RLNP | 0.0 | 33.0 | NE |
| Augmentation | - | 27.0 | NE |
| Augmentation followed by thyroplasty | - | 6.1 | NE |
| Clavien-Dindo ≥3b | 0.0 | 0.0 | NE |
| **Cardiac** | 16.9 | 20.9 | 5.7 (-4.1 to 15.5) |
| Clavien-Dindo ≥3b | 0.6 | 0.9 | NE |
| **Urological** | 2.9 | 8.7 | 3.9 (-1.9 to 9.7) |
| Clavien-Dindo ≥3b | 0.0 | 0.0 | NE |
| **Thromboembolic** | 4.1 | 1.7 | NE |
| Clavien-Dindo ≥3b | 0.0 | 0.0 | NE |
| **Infections** | 9.3 | 9.6 | -3.2 (-10.8 to 4.4) |
| Clavien-Dindo ≥3b | 0.0 | 0.9 | NE |
| **Wound / Diaphragm** | 0.6 | 3.5 | NE |
| Clavien-Dindo ≥3b | 0.0 | 1.7 | NE |
| **Chyle leak** | 4.1 | 3.5 | NE |
| Clavien-Dindo ≥3b | 0.6 | 1.7 | NE |
| **Other** | 7.0 | 6.1 | 0.7 (-5.9 to 7.3) |
| Clavien-Dindo ≥3b | 0.0 | 1.7 | NE |
|  |  |  |  |
| **CCI whole groups patients (mean)** | 16.0 | 26.4 | 10.7 (5.4 to 16.0) |
| **CCI whole groups patients, without RLNP (mean)** | 16.0 | 23.0 | 6.4 (1.6 to 11.1) |

**Table S3:** Values are in % unless otherwise indicated. Clavien–Dindo ≥3b percentages are calculated within each complication category (i.e., proportion of cases of that complication meeting ≥3b criteria). § Defined by the Esophagectomy Complications Consensus Group. MICE, Minimally Invasive transCervical oEsophagectomy; TMIE, Transthoracic Minimally Invasive oEsophagectomy; RLNP, recurrent laryngeal nerve palsy; CCI, Comprehensive Complication Index; RD, Risk Difference; MD, Mean Difference; CI, Confidence Interval; NA, Not Applicable; NE, Not Estimable. Cells labelled 'NE' could not be fitted by the doubly robust (AIPW) model. This occurs when the outcome shows too little variation to model, that is, no events in either group (for example 0 of 172 versus 0 of 115) or events in nearly all patients, or when events are too sparse in one of the groups to support a stable estimate.

| **Table S4. Comparison of perioperative outcomes and (re)admission rates between MICE and MIE after doubly robust (AIPW**, **augmented inverse probability of treatment weighting) analysis.** | | | |
| --- | --- | --- | --- |
| **Parameter** | **TMIE** | **MICE** | **RD / MD (95% CI)** |
|  |  |  |  |
| **Perioperative outcomes** |  |  |  |
| Duration of procedure (min), mean | 320.0 | 451.2 | 133.6 (112.3 to 154.8) |
| Operative time (min), mean | 248.5 | 362.3 | 112.3 (96.9 to 127.7) |
| Blood loss (mL), mean | 149.4 | 155.4 | 48.2 (-92.2 to 188.6) |
| Conversion to open (%) | 2.9 | 3.5 | NE |
| **Oncological outcomes** |  |  |  |
| Resected lymph nodes, mean | 28.0 | 31.7 | 4.5 (2.3 to 6.6) |
| Intrathoracic - high  paratracheal, along the recurrent laryngeal nerve and aorto-pulmonary window (106, 106recR, 106recL, 106tbL) | 14.5 | 97.4 | 75.9 (66.1 to 85.7) |
| Intrathoracic - low  Subcarinal (107) | 97.0 | 100.0 | NE |
| Paraesophageal (108, 110) | 99.4 | 100.0 | NE |
| N1 lymph node stations of the stomach (at least 3 of 6 node stations (1, 2, 3, 7, 8a, 8p, 9, and 11)) | 99.4 | 100.0 | NE |
| N2 lymph node stations of the stomach (at least 3 of 6 node stations) | 98.2 | 97.3 | -1.4 (-5.2 to 2.4) |
| Distant lymph node stations | 5.4 | 8.8 | 3.7 (-2.6 to 10.0) |
| Other (e.g. cervical) | 0.6 | 5.6 | NE |
| Positive lymph nodes (n), mean | 2.6 | 2.2 | -0.1 (-0.9 to 0.7) |
| Radicality (%) |  |  |  |
| R0 resection | 95.9 | 94.8 | -6.0 (-17.2 to 5.2) |
| R1 resection | 2.9 | 1.7 | NE |
| Not assessable | 0.6 | 0.9 | NE |
| **(Re)admission** |  |  |  |
| LOS, hospital (days), mean | 12.1 | 12.5 | 0.5 (-2.5 to 3.6) |
| LOS, ICU (days), mean | 2.3 | 2.7 | 0.5 (-1.1 to 2.0) |
| Readmission within 30 days (%) | 12.4 | 19.1 | 5.7 (-3.7 to 15.1) |

**Table S4:** TMIE, Transthoracic Minimally Invasive oEsophagectomy; MICE, minimally invasive transCervical oEsophagectomy; RD, Risk Difference; CI, Confidence Interval; MD, Mean Difference; ICU, intensive care unit; LOS, length of stay. NE, not estimable. Cells labelled 'NE' could not be fitted by the doubly robust (AIPW) model. This occurs when the outcome shows too little variation to model, that is, no events in either group (for example 0 of 172 versus 0 of 115) or events in nearly all patients, or when events are too sparse in one of the groups to support a stable estimate.
